# Cardiovascular health and proximity to urban oil drilling in Los Angeles, California

**DOI:** 10.1038/s41370-023-00589-z

**Published:** 2023-08-08

**Authors:** Jill E. Johnston, Arbor J. L. Quist, Sandy Navarro, Shohreh F. Farzan, Bhavna Shamasunder

**Affiliations:** 1 Division of Environmental Health, Department of Population & Public Health Sciences, Keck School of Medicine, University of Southern California, Los Angeles, CA USA; 2 LAGrit Media, Los Angeles, CA USA; 3 Department of Urban & Environmental Policy, Occidental College, Los Angeles, CA USA

**Keywords:** Oil and gas, Blood pressure, Environmental justice, Cardiovascular

## Abstract

**Background:**

Although ~18 million people live within a mile from active oil and gas development (OGD) sites in the United States, epidemiological research on how OGD affects the health of nearby urban residents is sparse. Thousands of OGD sites are spread across Los Angeles (LA) County, California, home to the largest urban oil production in the country. Air pollution and noise from OGD may contribute to cardiovascular morbidity.

**Objective:**

We examined the association between proximity to OGD and blood pressure in a diverse cohort of residents in LA.

**Methods:**

We recruited residents in South LA who lived <1 km from an OGD site. We collected three blood pressure measurements for each participant and used the second and third measurements to calculate averages for systolic blood pressure (SBP) and diastolic blood pressure (DBP) separately. We conducted multivariable linear regression to examine the relationship between distance to OGD sites and continuous SBP and DBP, adjusting for BMI, smoking status, distance to freeway, sex, age, and use of antihypertension medications, with a random effect for household. We examined effect measure modification by BMI category and smoking category.

**Results:**

Among the 623 adult participants, we found that for every 100 meter increase in distance from the OGD site, DBP was reduced by an average of 0.73 mmHg (95% CI: −1.26, −0.21) in this population. We observed stronger effects of proximity to OGD site on DBP among never smokers and among participants with a healthy BMI. The associations observed between proximity to OGD site and SBP were weaker but followed the same patterns as those for DBP.

**Impact:**

Our study suggests that living near urban oil drilling sites is significantly associated with greater diastolic blood pressure in urban Los Angeles communities. This research improves understanding of impacts from living nearby drilling operations on the health and welfare of this community, which is critical to inform public health relevant strategies.

## Introduction

In response to a drive for energy independence coupled with demands for fossil fuels, domestic oil and gas production in the United States (US) has surged over the last decade [1]. Across the continental US, there are over 1 million onshore oil and gas wells with an estimated 18 million people living <1600 m from an active oil extraction site in the US [2]. Increasingly, petroleum extraction is occurring in densely populated areas, and yet, epidemiological research on the health consequences for nearby urban residents is sparse [3–6]. California (CA), together with Texas, North Dakota, and Alaska account for ~60% of all oil produced domestically. Public health concern has accompanied this rapid growth in oil production [7].

Los Angeles (LA) County, CA, is home to one of the most petroleum-dense basins in the world, with thousands of extraction wells spread across multiple oil fields in 70 different communities [8, 9]. Land development, population growth, and oil exploration in LA occurred concurrently, leaving a patchwork of thousands of oil wells operating in very close proximity to homes, schools and parks [10]. Approximately 1/3rd of the 10 million LA County residents live less than 1 mile from an active oil or gas extraction site, and over 500,000 residents live less than 400 m away [10]. A single well typically operates for decades with neighbors facing impacts from construction, production, processing and transportation. Chemicals associated with oil extraction and production include carcinogens, mutagens, reproductive toxins, irritants and endocrine disruptors [4, 11–13]. These compounds can enter the nearby environment through spills, leaks, volatilization, and disposal [4, 11–14]. Research in California has identified increase air pollution, adverse birth outcomes and decreased respiratory function within 1 km of well sites [15–17].

Recent research demonstrates multiple health-hazardous air pollutants associated with petroleum extraction, including particulate matter (PM), nitric oxides (NOx), and volatile organic compounds (VOCs) including polyaromatic hydrocarbons, benzene, naphthalene, xylenes, toluene, ethylbenzene and formaldehyde [18–20]. Exposure to environmental stressors generated by OGD, such as air pollution, noise, and psychosocial stress, have been shown to individually and jointly contribute to cardiovascular morbidity and mortality in other environmental contexts [21]. Prior research has shown that inhalation of air pollutants can result in several adverse cardiovascular responses, including elevations in blood pressure (BP) [22, 23]. Elevated blood pressure is a leading risk factor for the global burden of disease and is strongly and directly related to cardiovascular disease (CVD), which remains the leading cause of morbidity and mortality in the United States [24, 25]. Epidemiological studies have consistently found that PM_2.5_ ambient air pollution is associated with a small, yet significant, increase in blood pressure [26–28]. While less widely studied, there is evidence of an association between exposures to VOCs with cardiovascular mortality, cardiovascular disease, and higher blood pressure [29, 30].

Although OGD generates multiple exposures that have been consistently linked to cardiovascular health risks, few studies have examined OGD impacts on blood pressure on nearby residents. Epidemiological studies have observed a positive association between proximity to OGD and prevalence of cardiology inpatient hospital admission in rural Pennsylvania [31]. A cross-sectional study in rural Colorado measured higher blood pressure among adults living in areas with higher density of wells compared to those farther away [32]. In a diverse cohort of residents living near urban oil drilling in Los Angeles, we examined the association between proximity to OGD and blood pressure.

## Methods

This study used a community-based participatory research approach to recruit from neighborhoods atop the Las Cienegas oilfield situated in South Los Angeles, CA as one component of the Health and Air Pollution Study [17]. These are densely populated neighborhoods of predominantly low-income Black and Latinx/Hispanic families facing disproportionate burden of environmental hazards [33, 34]. Participants were drawn from residents living <1 km from either (1) a well site with 28 wells that was actively producing oil during the entire study period; or (2) a site housing 21 wells which were idle (i.e., not actively producing any oil or gas) during the study period, but had previously been active for decades. To be eligible for the Health and Air Pollution study, participants were at least 9 years old, spoke English, Spanish or Korean, and lived within 1000 m of one of the oil sites of interest for at least 2 years. The research was a community-academic collaboration with local community health workers together with Esperanza Community Housing and Redeemer Community Partners, both community-based organizations working in the neighborhoods. Details on the recruitment methods are available elsewhere [17]. The study was approved by the University of Southern California Institutional Review Board. Participants who provided written consent completed a baseline demographic and health questionnaire and provided physiological measurements.

### Health questionnaire

A questionnaire was administered in the participant’s preferred language (Spanish, English or Korean) and participants were asked sociodemographic information, race/ethnicity, sex, age, tobacco exposure (e.g., smoking history, current smoking practices, presence of indoor environmental tobacco smoke), occupation, medication use and residential history. We collected information about disease history, including if the participant ever had a doctor-diagnosis of hypertension. We reviewed the participant-reported medication usage and identified participants who were currently taking anti-hypertensive medications.

### Cardiovascular health and physiological measurements

For each adult participant, we collected 3 blood pressure measurements using an Omron 705IT (Omron Corporation, Kyoto, Japan) [35] by trained study staff. To obtain these measures, an adjustable, inflatable cuff was fitted to a participant’s dominant arm, which was extended onto a flat surface, ensuring that the bend in the elbow was at heart level with feet flat on the floor. The participant rested 5 min before we collected three measurements, each 1 min apart. The reported systolic blood pressure (SBP) and diastolic blood pressure (DBP) is the average of the 2nd and 3rd measurements. Each participant’s height (to nearest 0.1 cm) and weight (0.1 kg) were measured and used to calculate and categorize participant body mass index (BMI, underweight: <18.5 healthy: 18.5–24.9, overweight: 25–29.9, obese: 30+, according to the categories from the National Heart, Lung, and Blood Institute). Due to the small number of underweight participants, these participants were combined with the healthy BMI category for analysis. The measurements were shared back with the participants at the end of the visit along with clinic guidelines to aid interpretation of the results.

### Exposure metrics

The location and status of the wells was retrieved from the California Geologic Energy Management Division (CalGEM) for the study period. Participant addresses were geocoded and the Euclidean distance to the well sites and freeways was calculated. Daily PM_2.5_ concentrations were estimated for the day of the study visit based on the Los Angeles North Main Street monitor, located ~4–5 miles northeast from the study area and operated by South Coast Air Quality Management District.

### Statistical analysis

We conducted exploratory data analysis, examining participant characteristics by hypertensive status (based on participant-reported doctor-diagnosed hypertension), proximity to OGD sites, and variable distributions. We found continuous SBP and DBP to approximate a normal distribution and proceeded with untransformed variables for subsequent analyses. We conducted multivariable linear regression to assess the relationship between distance to OGD sites and continuous SBP and DBP, adjusting for BMI category (healthy, overweight, obese), use of anti-hypertensive medications, smoking status (current, former, never), distance to freeway (<200 m from highway vs. ≥200 m from highway), sex, and age (continuous) [36]. As participants could live in the same household as other participants, we conducted all analyses with a random effect for household. We examined effect measure modification by BMI category, smoking category, and race/ethnicity.

### Secondary analyses

Given that approximately one-fifth of participants (*n* = 129) reported taking anti-hypertensive medications, we further explored this relationship using adjusted blood pressure measurements for participants on anti-hypertensive medications by adding 15 mmHg to the SBP values and 10 mmHg to DBP values, based on methods from prior research [36–38]. We also conducted analyses where we excluded participants taking anti-hypertensive medication and participants who reported doctor-diagnosed hypertension. Because PM_2.5_ has been acutely associated with BP [26], we adjusted for PM_2.5_ daily average on the day BP was assessed. Finally, we examined the association between distance to OGD site and the presence of measured stage 1 hypertension, defined as SBP > 130 mmHg or DBP > 80 mmHg [39]. All analyses were conducted in R 4.1.0.

## Results

A total of 665 adult residents (at least 18 years of age) participated in this neighborhood study to measure physical health symptoms from 488 distinct addresses [17]. Eleven participants were subsequently excluded for living outside of the study area after confirmation of residential address, 8 participants were excluded for outlier BP measurements (systolic BP > 180 mm Hg or diastolic BP > 120 mm Hg, which may reflect error in cuff size fitting or placement), and 16 participants did not complete BP measurements. Seven additional participants were excluded from analysis because of missing key covariates (sex and or height). This resulted in a total population of 623 individuals. The mean age of the participants was 49 years (range 18–85) with 22.6% of participants over the age of 65. The majority were female (66.8 %) and all participants identified as people of color (Black, Latinx/Hispanic, Asian or multi-racial) (Table 1). Forty-one percent of participants were considered obese, reflecting the general rates of obesity in South LA [40]. Eight percent were current smokers. On average, participants had lived in the neighborhood for 19 years. The median distance between the home and the OGD site was 260 meters (range: 39–970 m). The mean SBP and DBP measurements for the analysis cohort were 124.7 ± 18.6 and 79.5 ± 11.1 mmHg, respectively. Approximately 57% of the cohort met the criteria of state 1 hypertension (average measure >130 mmHg SBP or >80 mmHg DBP).

**Table 1:** Characteristics of adult study cohort, overall and categorized by distance to well (median split at 260 m).

Characteristic	Overall (*N* = 623)	<260 m from well (*N* = 310)	≥260 m from well (*N* = 313)	*p*
Sex (%)				
Female	416 (66.8)	211 (68.1)	205 (65.5)	0.551
Male	207 (33.2)	99 (31.9)	108 (34.5)	
Age category (%)				
18–34	142 (22.8)	68 (21.9)	74 (23.6)	0.005
35–64	340 (54.6)	155 (50.0)	185 (59.1)	
≥65	141 (22.6)	87 (28.1)	54 (17.3)	
Race/ethnicity (%)				
Hispanic or Latino	457 (73.5)	218 (70.3)	239 (76.6)	<0.001
Asian	45 (7.2)	45 (14.5)	0 (0.0)	
Black or African American	89 (14.3)	37 (11.9)	52 (16.7)	
Multi-racial or Other Race	31 (5.0)	10 (3.2)	21 (6.7)	
BMI category (%)				
Underweight	8 (1.3)	5 (1.6)	3 (1.0)	0.03
Healthy	138 (22.2)	73 (23.5)	65 (20.8)	
Overweight	222 (35.6)	123 (39.7)	99 (31.6)	
Obese	255 (40.9)	109 (35.2)	146 (46.6)	
Smoking status (%)				
Never	431 (69.2)	217 (70.0)	214 (68.4)	0.279
Current	51 (8.2)	20 (6.5)	31 (9.9)	
Former	141 (22.6)	73 (23.5)	68 (21.7)	
Distance to freeway (%)				
<200 m	154 (24.7)	59 (19.0)	95 (30.4)	0.001
≥200 m	469 (75.3)	251 (81.0)	218 (69.6)	
Particulate matter (PM) 2.5, daily mean ± SD	13.9 ± 5.8	14.0 ± 5.6	13.9 ± 6.0	0.921
Report doctor-diagnosed hypertension (%)	194 (31.1)	106 (34.2)	88 (28.1)	0.121
Taking antihypertension medications	129 (20.7)	73 (23.5)	56 (17.9)	0.1
Systolic blood pressure, mmHg, mean ± SD	124.7 ± 18.6	125.4 ± 18.8	124.0 ± 18.5	0.35
Diastolic blood pressure, mmHg, mean ± SD	79.5 ± 11.1	80.0 ± 11.4	79.0 ± 10.9	0.291
Hypertension 1 during study visit (%)	354 (56.8)	186 (60.0)	168 (53.7)	0.13

Categorical variables were compared between the distance to well groups using a chi-squared test with continuity correction and continuous variable were compared using *t*-test.

**Fig. 1:**
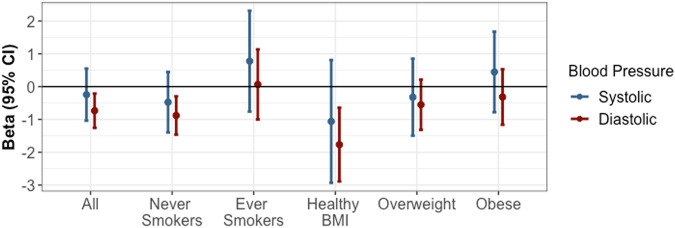
Proximity to oil and gas development (OGD) site and blood pressure by subgroup. Association between systolic and diastolic blood pressure (mmHg) and distance from the OGD site (per 100 m) among all adult participants (adjusted for BMI, smoking status, distance from freeway, sex, age and use of anti-hypertensive medication, with a household random effect) and stratified by smoking status and BMI category.

In multivariable linear models, we observed that greater distance from the oil and gas wells was associated with lower blood pressure, on average, when adjusting for BMI, smoking status, distance from freeway, sex, age and use of anti-hypertensive medication. This finding was statistically significant for DBP, showing that for every 100 m increase in residential distance from the OGD site, DBP was reduced by an average of 0.73 mmHg (95% CI: −1.26, −0.21) in this population (Fig. 1 and Table S1). The effect was smaller and not statistically significant for SBP, with a reduction by 0.24 mmHg (95% CI: −1.04, 0.55) per 100 m increase in distance. The results were consistent when including the outlier measurements (Table S2).

We stratified the analysis considering two risk factors for elevated blood pressure: smoking status and BMI (Fig. 1). We observed stronger effects of distance on blood pressure among the never smokers, with an increase of 100 m in distance from the OGD site associated with, on average, a 0.48 mmHg (95% CI −1.40, 0.44) and 0.88 mmHg (95% CI −1.46, −0.30) decrease in SBP and DBP, respectively. Among the participants with a healthy BMI (BMI < 25 based on measured height and weight), we observed a reduction of 1.06 mmHg (95% CI −2.93, 0.81) and 1.77 mmHg (95% CI −2.89, −0.64) in SBP and DBP respectively for each 100 m increase in residential distance from the ODG site. When analyses were stratified by race/ethnicity, we observed a strong, albeit imprecise, impact of distance on blood pressure among Black participants, with an increase of 100 m in distance from OGD site associated reduction of 0.21 mmHg (95% CI −3.14, 2.73) and 1.62 mmHg (95% CI −3.62, 0.38) in SBP and DBP, respectively (Table 2). Among female participants, we observed lower SBP and DBP at greater distances from well site increased and little change among male participants (Table 2).

**Table 2:** Sensitivity analyses examining the relationship of between systolic and diastolic blood pressure and distance to the OGD site (continuous, per 100 m).

Model	Beta (95% CI)	
Sensitivity analyses	SBP, mmHg	DBP, mmHg
Adults not reporting hypertension (*N* = 429)	−0.27 (−1.10, 0.55)	−0.73 (−1.28, −0.17)
Adults not on hypertension medication (*N* = 494)	−0.13 (−0.98, 0.73)	−0.59 (−1.16, −0.03)
Adjusted for antihypertension medication by +10/15 SBP and DBP	−0.31 (−1.19, 0.56)	−0.78 (−1.33, −0.22)
All adjusted for daily PM_2.5_ concentration	−0.25 (−1.05, 0.54)	−0.74 (−1.26, −0.21)
Stratified by race/ethnicity		
Black or African American (*N* = 89)	−0.21 (−3.14, 2.73)	−1.62 (−3.62, 0.38)
Hispanic or Latinx (*N* = 457)	−0.35 (−1.18, 0.49)	−0.69 (−1.23, −0.14)
Stratified by sex		
Female (*N* = 416)	−0.24 (−1.20, 0.71)	−0.97 (−1.56, −0.38)
Male (*N* = 207)	−0.05 (−1.45, 1.35)	−0.13 (−1.13, 0.87)

### Sensitivity analysis: ambient air pollution

As fine particulate matter pollution (PM < 2.5 µm in diameter) may adversely impact blood pressure, we considered daily PM_2.5._ concentration estimates on the day of the measurement for all participants. However, we found adjusting for daily PM_2.5_ concentrations did not change the results of the association between proximity and blood pressure measurements (Table 2).

### Hypertension and proximity to oil and gas development sites

When assessing the presence of stage 1 hypertension with respect to living near (<260) or farther (260–1000 m) from the well site, we observed higher odds of hypertension (OR = 1.49, 95% 1.02, 2.18) among the residents living near the OGD site. The findings were similar when considering various restrictions and sensitivity analyses (Table 3). We observe a small, yet significant decrease in the odds of hypertension among participants for every 100 m increase in distance away from the well site.

**Table 3:** Association between hypertension (>130 systolic or >80 diastolic) and distance to the OGD site among adult participants, adjusting for BMI category, smoking status, freeway distance, sex, antihypertension medications, age, and with a random effect for household.

Exposure	OR (95% CI)
Near to well site (<260 m from well) (*N* = 623)	1.49 (1.02, 2.18)
Restricted to those not reporting hypertension (*N* = 429)	1.61 (1.03, 2.51)
Restricted to those not on hypertension medication (*N* = 494)	1.60 (1.04, 2.47)
All participants, no antihypertension medication covariate	1.50 (1.03, 2.18)
Continuous distance to well (per 100 m)	0.87 (0.77, 0.97)

## Discussion

This study contributes to the growing literature on the health consequences for urban residents living near oil and gas extraction. Among a multiethnic cohort living atop the Las Cienegas oil field in South Los Angeles, we identified that residents living closer to the OGD site have, on average, higher blood pressure and face higher risk of stage 1 hypertension compared with residents that live farther away. This effect was more pronounced among never smokers, those within the healthy BMI category, and women residents. The association between distance to the oil well site and blood pressure was more apparent in non-smoking participants and those with lower BMI, potentially because greater BMI and smoking are known risk factors for elevated blood pressure. This study provides additional evidence of potential adverse relationship between cardiovascular health and oil drilling activities in an urban context.

Prior studies have found evidence of adverse impacts of oil and gas extraction on cardiovascular health. Communities near OGD and oil refineries are reported to have higher risks of hypertension compared with other communities farther away from these exposures [41]. A study among residents in rural Colorado found the highest SBP and DBP among people near the most OGD activity who were not taking prescription medications [32]. McKenzie and colleagues observed ~5 mmHg increase in SBP and 4 mmHg increase in DBP when the highest OGD exposed group was compared to the lowest OGD exposed group, after considering age and sex. Exposure was based on an oil and gas intensity metric within 16 km of the home and therefore not directly comparable to the metric used in this analysis. Nonetheless, we observe a similar trend among both rural residents of Colorado and urban LA, such that residents more exposed to OGD have, on average, greater blood pressure. Similarly, a study in the Niger Delta found residents chronically exposed to oil extraction and gas flaring had statistically higher SBP and DBP than the unexposed counterparts [42]. A randomized cross-sectional survey in the same region found that adults living in rural areas with OGD were nearly 5 times as likely to report hypertension compared to those living away from OGD after adjusting for socioeconomic and lifestyle factors [43].

Occupational studies suggest an association between crude oil exposures and cardiovascular health [44]. Longitudinal studies of oil cleanup workers have found duration of cleanup work associated with increased risk of myocardial infarction [45–47]. Additionally, the studies also found that living in proximity to the oil spill was associated with heart disease when compared to individuals living farther away [45, 46]. Among oil spill workers after the *Deepwater Horizon* disaster, SBP and DBP were higher with increased levels of total petroleum hydrocarbon exposure, with the strongest trend for DBP [44]. Further, higher exposure to total hydrocarbons was associated with elevated risk of newly detected hypertension, especially among workers with obesity and those who identified as Black, current smokers, and male [44]. Racism and poverty are associated with various stressors, including unemployment, financial stress, and violence, that may increase the risk of hypertension.

Oil and gas extraction sites and on-site operations typically produce a complex mixtures of air pollutants, including hydrocarbons such as benzene and diesel particulate matter [4, 11–13]. Hazardous compounds can be volatilized or aerosolized during extraction via active evaporating pits, flares, surface spills, processing, and transportation [12]. Research in communities atop the Las Cienegas oil field identified volatilized hydrocarbons were affecting air quality throughout the adjacent neighborhoods [19, 20] and revealed episodic peaks of air toxics likely attributable to local oil and gas operations [48]. Exposure to ambient volatile organic compounds (VOCs) has been associated with adverse cardiovascular outcomes such as emergency department visits for heart failure [49–51] and hypertension [52]. Additionally, oil drilling has been associated with emissions of toxic metals such as manganese and nickel [53, 54], and exposure to these metals may increase risk for hypertension [36, 55–59]. There may be direct impacts of environmental contaminants on biological and physiological processes, as well as psychosocial effects of living in close proximity to an OGD site. The mechanism underlying the association between OGD exposure and blood pressure, however, is still unclear. Exposure to toxic pollution and stress related to fear of potential impacts of disasters may increase the health burden in these communities, as environmental justice communities not only face additional burdens due to toxic releases, but often lack the social or financial resources to mitigate the exposures [60]. Particulate matter, noise, and stress from OGD can activate the sympathetic nervous system in humans, leading to greater oxidative stress and systemic inflammatory responses [32, 61, 62]. This may lead to autonomic nervous imbalance and endothelial dysfunction, which can then contribute to hypertension [22].

To our knowledge, this is the first study to examine the relationship between cardiovascular health in diverse urban communities and oil well sites. To date the health research OGD in the US is largely based in rural and majority non-Hispanic White communities. Our study involves a predominantly low-income community of color living in an historically underserved, environmental justice community. In this study we identify proximity to urban oil and gas development sites as a factor associated with greater blood pressure. While both SBP and DBP showed decreases with increasing distance from the well, only DBP was statistically significant. Though similar trends are observed for both SBP and DBP, it is possible that exposure to pollutants from OGD sites may target mechanisms that preferentially impact DBP, such as autonomic function, vascular reactivity, and vasoconstriction [60]. Similarly, other studies on air pollution and blood pressure in adults have reported effects limited to DBP [21, 63, 64].

Environmental justice communities can face chemical and non-chemical stressors that can pose potential cumulative and interrelated consequences for blood pressure [65]. While limited by a cross-sectional design, our study presents novel findings linking cardiovascular health effects to urban oil drilling. We cannot rule out potential confounding by unmeasured covariates or differential participation rates based on concerns about neighborhood health or environmental quality. We cannot account for lifetime residential history, individual household characteristics nor occupational exposures. Proximity is used as a proxy for exposure to pollution associated with the well sites and may represent more than just oil-related exposure (e.g., noise, stress) that cannot be disentangled from chemical exposures. Future work will include assessing neighborhood scale air pollution to better understand potential spatiotemporal patterns of regional, freeway and oil drilling related exposures. The study of environmental hazards and human exposures in nearby communities remains valuable information for public health protection, pollution prevention, and exposure reduction. The results of research can fill gaps in government data available at a local level and draw attention to local environmental health hazards.

## Conclusions

Together, our findings suggest that living near urban oil drilling sites is significantly associated with greater diastolic blood pressure in South Los Angeles. This research improves understanding of impacts from living nearby drilling operations on the health and welfare of this community, which is critical to inform public health relevant strategies. As a community of predominantly low-income residents of color, these impacts raise environmental justice concerns about the effects of urban oil drilling. Reducing emissions, increasing the distance between oil operations and residents, and investments in renewable energy and energy efficiency measures that reduce reliance on fossil fuels overall—could protect the cardiovascular health of residents near oil wells.

### Supplementary information

Reporting Checklist

Supplemental Information

## Data Availability

The survey data that support the findings of this study are not publicly available to safeguard the privacy of the participants and to maintain trust with affected communities. Data may be available from the authors upon reasonable request and with permission of the University of Southern California Institutional Review Board. Location and production of oil and gas wells is available through the California Geologic Energy Management Division.
