# Supplementary material for: Cardiovascular health and proximity to urban oil drilling in Los Angeles, California
Source: J Expo Sci Environ Epidemiol. 2023 Aug 8;34(3):505–11. doi: 10.1038/s41370-023-00589-z (PMC10850428; doi:10.1038/s41370-023-00589-z)
Supplement: Supplementary file 2 — Supplemental Information [file 41370_2023_589_MOESM2_ESM.docx]

Cardiovascular Health and Proximity to Urban Oil Drilling in Los Angeles, California

Jill E Johnston^1*^, Arbor JL Quist^1^, Sandy Navarro^2^, Shohreh F Farzan^1^, Bhavna Shamasunder^3^

^1^ Division of Environmental Health, Department of Population & Public Health Sciences, Keck School of Medicine, University of Southern California

^2^ LAGrit Media

^3^ Department of Urban & Environmental Policy, Occidental College

**Supplemental Information**

**Table S1.** Association between systolic and diastolic blood pressure (mmHg) and distance from the OGD site (per 100 m) among all adult participants (adjusted for BMI, smoking status, distance from freeway, sex, age and use of hypertensive medication, with a household random effect) and stratified by smoking status and BMI category (same results as displayed in Figure 1).

| **Subset** | **SBP, mmHg** | **DBP, mmHg** |
| --- | --- | --- |
| All Participants (N=623) | -0.24 (-1.04, 0.55) | -0.73 (-1.26, -0.21) |
| Ever Smokers (N=192) | 0.78 (-0.76, 2.31) | 0.07 (-1.00, 1.13) |
| Never Smokers (N=431) | -0.48 (-1.40, 0.44) | -0.88 (-1.46, -0.30) |
| Healthy BMI (N=146) | -1.06 (-2.93, 0.81) | -1.77 (-2.89, -0.64) |
| Overweight (N=222) | -0.32 (-1.49, 0.85) | -0.55 (-1.32, 0.21) |
| Obese (N=255) | 0.45 (-0.78, 1.67) | -0.32 (-1.16, 0.53) |

**Table S2.** Association between systolic and diastolic blood pressure (mmHg) and distance from the OGD site (per 100 m) among all adult participants (adjusted for BMI, smoking status, distance from freeway, sex, age and use of hypertensive medication, with a household random effect) and stratified by smoking status and BMI category, without any outlier exclusion for blood pressure.

| **Subset** | **SBP, mmHg** | **DBP, mmHg** |
| --- | --- | --- |
| All Participants (N=631) | -0.24 (-1.09, 0.62) | -0.76 (-1.31, -0.20) |
| Ever Smokers (N=194) | 0.50 (-1.09, 2.09) | -0.11 (-1.22, 1.00) |
| Never Smokers (N=437) | -0.35 (-1.36, 0.66) | -0.85 (-1.49, -0.22) |
| Healthy BMI (N=149) | -1.51 (-3.71, 0.69) | -2.09 (-3.34, -0.83) |
| Overweight (N=224) | -0.34 (-1.52, 0.84) | -0.49 (-1.30, 0.32) |
| Obese (N=258) | 0.67 (-0.64, 1.98) | -0.31 (-1.18, 0.57) |

**Table S3**. Association between systolic and diastolic blood pressure (mmHg) and distance from the OGD site (per 100 m) stratified by site. Analyses include all adult participants (adjusted for BMI, smoking status, distance from freeway, sex, age and use of hypertensive medication, with a household random effect).

| **Subset** | **SBP, mmHg** | **DBP, mmHg** |
| --- | --- | --- |
| Active site (N=631) | -0.41 (-1.58, 0.76) | -0.53 (-1.32, 0.27) |
| Inactive site (N=194) | -0.03 (-1.38, 1.33) | -0.63 (-1.45, 0.18) |
